# Atopy and adenotonsillar hypertrophy in mouth breathers from a reference center

**DOI:** 10.5935/1808-8694.20130123

**Published:** 2015-10-08

**Authors:** Emanuel Capistrano Costa, Henrique Augusto Cantareira Sabino, Carolina Sponchiado Miura, Carolina Brotto de Azevedo, Ullissis Pádua de Menezes, Fabiana Cardoso Pereira Valera, Wilma Terezinha Anselmo-Lima

**Affiliations:** a MD; Resident Physician, University Hospital, School of Medicine of Ribeirão Preto, University of Sãc Paulo; b PhD; Professor, School of Medicine of Ribeirão Preto, University of São Paulo; c Senior Associate Professor; Associate Professor, School of Medicine of Ribeirão Preto, University of São Paulo

**Keywords:** adenoids, allergy and immunology, mouth breathing, tonsillar hypertrophy

## Abstract

Mouth breathers use the oral cavity as their principal breathing route. The main causes include: adenotonsillar hypertrophy and inflammatory diseases such as allergic rhinitis.

**Objective:**

To look for atopy, the main allergens involved and to check for atopy as a comorbidity with the degree of hypertrophy of the tonsils and adenoids in mouth breathers.

**Method:**

A historical cohort study with cross-sectional review of 308 medical charts of patients treated at a mouth breather care center of a tertiary hospital in the period of 2008–2010. We collected data on the mouth breather's clinical history and we ran otolaryngological exams, flexible nasal endoscopy and skin prick test to aeroallergens.

**Results:**

Of 308 patients, 36% were positive on allergy testing, with 95 % of atopic patients being positive for mites. Among all patients, 46% had adenoid hypertrophy; of these, 37% were atopic and 47% had tonsillar hypertrophy, and among these, 33% were atopic.

**Conclusion:**

We found no direct correlation between atopy and the degree of tonsils and adenoid hypertrophy observed among the mouth-breathing patients assessed. si.

## INTRODUCTION

Mouth breathing is a frequent complaint in pediatric and ENT outpatient wards, causing great discomfort to the patients' families, impairing their quality of life. Mouth breathing individuals replace nasal breathing for a mouth supplementation or a mixed breathing for longer than six months1., 2.. This condition has multiple causes, from nasal septum deviations to craniofacial deformities and tumors. The most frequent causes in pediatric patients are adenotonsillar hypertrophy, inflammatory and allergic diseases, such as allergic rhinitis - highly prevalent in the population3., 4.. Early diagnosis through an interdisciplinary approach is crucial for preventing facial growth and development disorders in mouth breathers.

Adenoid and tonsillar tissues are the main components of Waldeyer's ring, which may be enlarged by various causes, including infections by different microorganisms and allergies5., 6., 7.. The role played by allergy in promoting chronic inflammation with adenoid and tonsillar tissue hypertrophy in children remains unclear5., 8.. Atopy seems to play a different role in tonsils and adenoids. Tonsils have a thicker squamous epithelium, which hinders the penetration of antigens and thus suffer less of the inflammatory reaction caused by respiratory antigens. While the adenoid is located in a more limited space, in such a way that the edema caused by local inflammatory reaction gives rise to a matching greater airway obstruction^9^. Another factor that contributes to a higher action of allergens in the pharyngeal tonsils is the anatomical proximity of the nasal cavity to the nasopharynx, and the fact that these two regions have the same lymphatic drainage^6^. This study aimed to discuss the relationship between atopy and adenotonsillar hypertrophy in mouth breathers.

The objectives of this study are: to determine the frequency of atopy, adenotonsillar hypertrophy and the major allergens involved; and to investigate the correlation of atopy with the degree of adenotonsillar hypertrophy in mouth breathers evaluated in the Mouth Breather Care Center of a tertiary hospital.

## METHOD

We ran a cross-sectional cohort study in a tertiary care center for mouth breathing patients. We reviewed the medical records and filled out the research protocol of all the patients seen in our service in the period between 2008 and 2010. This study was approved by the Ethics Committee of our institution, under protocol number 127482. It was not necessary to fill out the informed consent form - a procedure approved by the Ethics in Research Committee, because this study used only data from medical records, all the patients involved did not have their identity disclosed.

Patient inclusion criteria were: age between 3 and 12 years, mouth breathing - assessed by the available clinical history and the availability of the following results in the medical chart: 1) Complete clinical ENT examination regarding tonsil size; 2) Assessment of patient sensitivity vis-à -vis airborne allergens by means of the prick test. 3) Fiberoptic endonasal examination to assess pharyngeal tonsil size.

The exclusion criteria were: patients who had associated diseases (genetic syndromes, dense deposit diseases, immunodeficiencies and craniofacial malformation); patients whose medical records did not have the necessary information for the study and patients already submitted to adenoid or tonsil surgery.

Upon admission, all patients were submitted to fiberoptic nasal endoscopy and the prick test, thus the data needed to complete the study protocol was obtained from the admission form. Of the 533 medical records reviewed, 50 were excluded because the patients had associated diseases (genetic syndromes, dense deposit diseases and immunodeficiencies), 127 because the medical records were incomplete and 48 did not meet the age criteria set for the study. Of the remaining 308 patients who entered the study, 183 were males and 125 were females. Their ages ranged between 3 and 12 years, with a mean of 7.3 years. There was no pairing between the atopic and non-atopic patient groups, except for having mouth breathing and matching the inclusion criteria.

We used the Brodsky scale^10^ to assess palatine tonsil size. On such scale: Grade I indicates that the tonsil blocks less than 25% of the airway; grade II 25–50%; 50–75% is grade III; and grade IV is associated with more than 75% obstruction. Patients with more than 50% of airway obstruction caused by the palatine tonsil (grades III and IV) were classified as having tonsillar hypertrophy.

Nasal endoscopy was performed with a 3.2 mm flexible fiberoptic scope, and we standardized the method of such examination, as well as adenoid size checking. For the pharyngeal tonsil assessment, the patients were divided into three groups. Group I: greater than 70% of cavum obstruction in at least one of the nostrils; Group II: 30% to 70% obstruction; and Group III: less than 30% obstruction of the cavum in both nostrils. Only those patients in Group I were classified as having adenoid hypertrophy.

The prick test was carried out using the following standardized allergen extracts from the ALK-Abelló laboratory: *Dermatophagoides pteronyssinus, Dermatophagoides farinae, Blomia tropicalis, Canis familiaris, Felis domesticus, Blatella germanica, Periplaneta americana, Alternaria alternata, Aspergillus fumigatus, Cladosporium hominis, Penicilium notatum*, pollens, negative control (saline solution) and positive control (histamine–1mg/ml). The technique used was standardized by the European Academy of Allergy and Clinical Immunology^11^. We punctured the volar surface of the forearm and read after 15 minutes, considering positive those skins papules with average diameters greater than 3 mm when compared to the negative control. We excluded patients with skin lesions, dermatographic urticaria, and those who used medications that could compromise the test result. Patients with positive skin test to at least one of the allergens tested were considered atopic.

We used the Fisher's method for contingency statistical analysis and a *p* less than 0.05 was considered statistically significant.

## RESULTS

**Graph 1:**
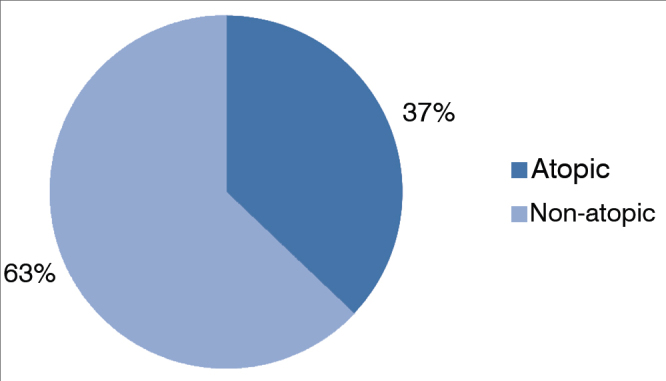
Incidence of atopy in mouth breathers.

**Graph 2:**
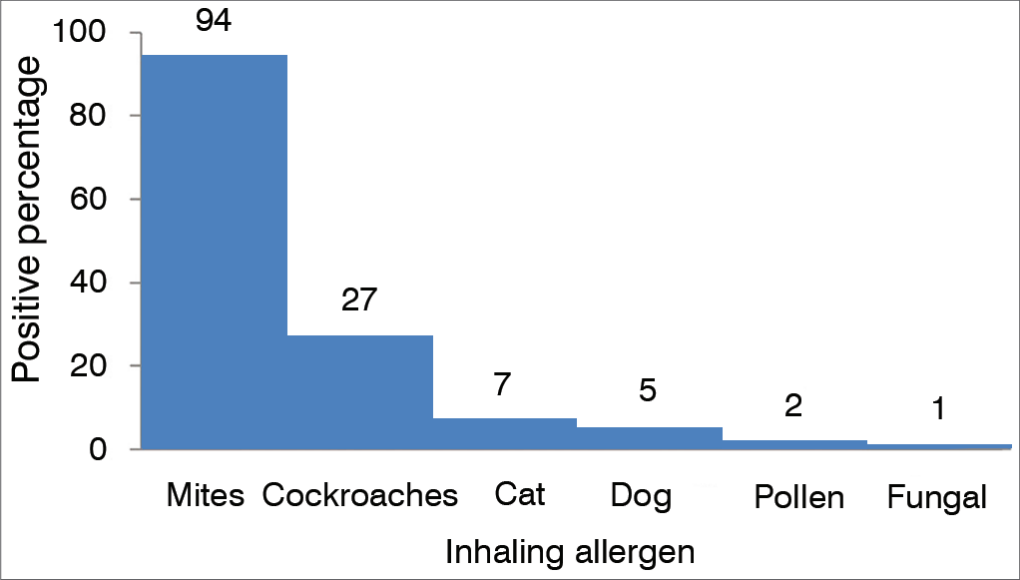
Response percentage of atopic patients to allergen extracts in skin prick tests.

Of the 308 mouth breathing patients selected, 110 (36 %) were atopic and had positive skin tests to inhalant allergens (Graph 1). Atopic patients had the following skin test results: 105 (95%) patients were positive for mite allergens, 26 (24%) for cockroaches, seven (6%) for cat fur, five (5%) for dog hair, three (2%) for pollen and only one patient was positive for fungal antigens (Graph 2).

**Graph 3:**
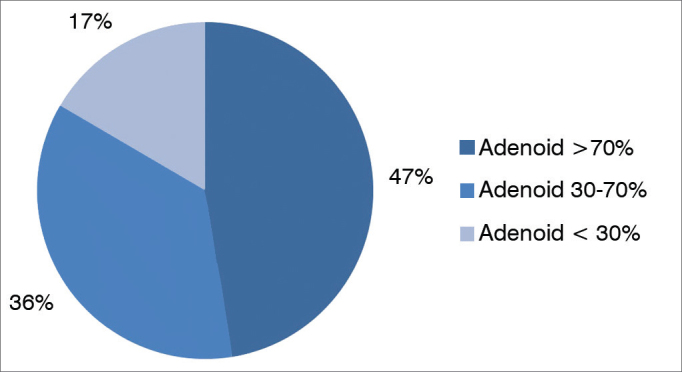
Mouth breathers distribution as to adenoid size.

Of the 308 mouth breathers assessed, 141 (46%) had adenoid hypertrophy, with the pharyngeal tonsil obstructing more than 70 % of the cavum, 111 (36%) had obstruction between 30–70% and only 56 (18%) had less than 30% obstruction of the cavum (Graph 3).

**Graph 4:**
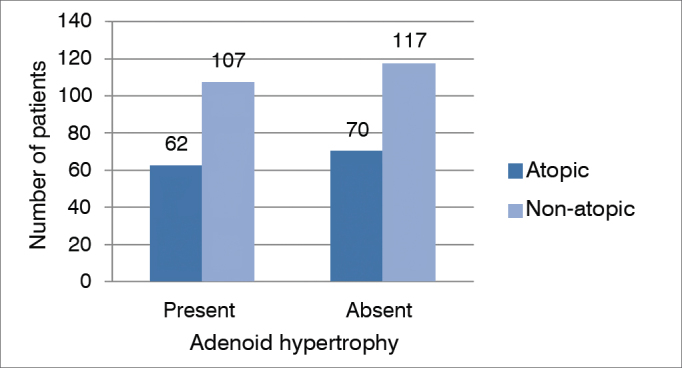
Relationship between adenoid hypertrophy and atopy in mouth breathers.

Of the 141 patients with adenoid hypertrophy, 52 (37%) were considered atopic by skin test and 89 (63%) were non-atopic; whereas patients without adenoid hypertrophy, 58 (35%) were atopic and 109 (65%) were not atopic. We carried out a statistical analysis using the Fisher's contingency test as to the prevalence of atopy in patients with adenoid hypertrophy, which yielded results without statistical significance (*p* = 0.7213) (Graph 4).

**Graph 5:**
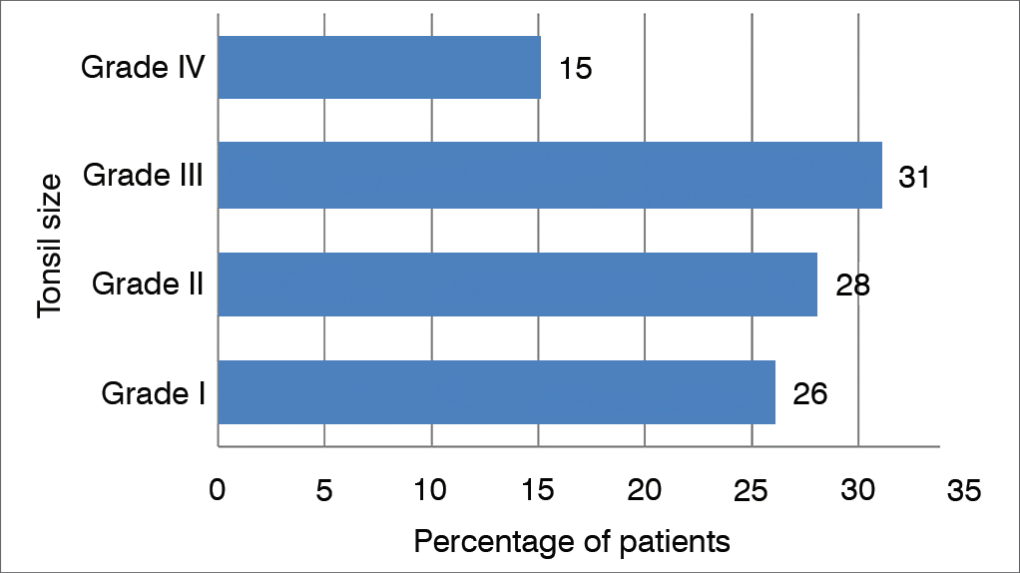
Mouth breathers distribution according to palatine tonsil size.

Also, from the 308 patients with mouth breathing, 146 (47%) were considered with tonsillar hypertrophy, 48 (15%) with grade IV tonsils and 98 (32%) with grade III tonsils. In the remaining 162 patients we noticed: 82 (27%) with grade II tonsils and 80 (26%) with grade I tonsils (Graph 5).

Of the 146 patients with tonsillar hypertrophy, 48 (33%) were considered atopic by the skin test and 98 (67%) were non-atopic; whereas in patients without tonsillar hypertrophy, 62 (38%) were atopic and 100 (62%) were not atopic. We did the statistical analysis using the Fisher's contingency method as to the prevalence of atopy in patients with tonsillar hypertrophy, which did not show statistically significant results (*p* = 0.3426) (Graph 6).

**Graph 6.6:**
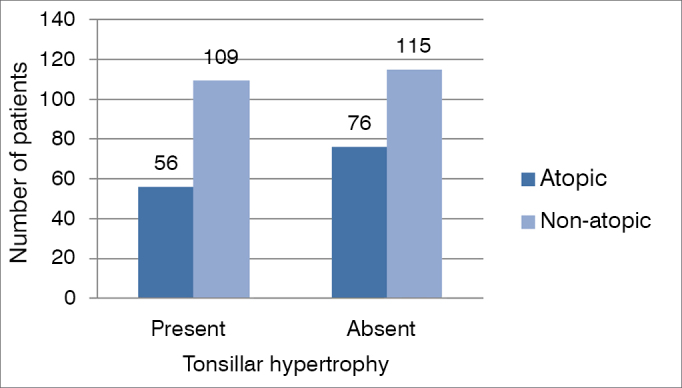
Relationship between tonsillar hypertrophy and atopy in mouth breathers.

Of the 308 mouth breathers assessed, 80 had adenotonsillar hypertrophy, whereas 61 had only adenoid hypertrophy and 66 only tonsil hypertrophy. Also, from the total, 101 (33%) had no increase in lymphoid tissues and 207 (67%) had at least one hypertrophic tissue among all the ones considered.

## DISCUSSION

We evaluated the frequency of atopy in patients with mouth breathing; 36% had positive skin tests to inhalant antigens, which is in agreement with other studies. Barros et al.^1^ evaluated 140 mouth breathing patients and found atopy in 44% of the subjects. This incidence is higher than its mean value found in the normal population - in which sensitization to allergens can vary from 10 to 30%^12^. Sadeghi-Shabestari et al.^6^ found 70% positive skin reactions to aeroallergens and food antigens in a group of 117 children with adenotonsillar hypertrophy and only 10% were positive in a control group of children of the same age, but without adenotonsillar hypertrophy. For the authors, allergic diseases and sensitization to different allergens are risk factors for adenotonsillar hypertrophy in children, and allergic rhinitis is among the principal causes of mouth breathing, since their incidence in these patients is substantially increased1., 2., 5., 6., 13., 14..

Regarding the profile of allergens tested in the atopic patients, the highest rate of sensitization was seen to allergens of mites (95%) followed by cockroach allergens (24%) - and these results were similar to the findings reported by Barros et al.^1^ and Santos et al.^15^ in the Brazilian population, in Ribeirão Preto and in Belo Horizonte, respectively. Allergen prevalence data may vary according to the population studied, our sample was obtained from the countryside of the state of São Paulo, then similar to the two studies cited. Huang & Giannoni^16^, in the USA, studied 315 children with adenoid hypertrophy associated with allergic rhinitis and compared them with a control group of 315 children with allergic rhinitis only. The authors reported that only the sensitization to fungal allergens was considered a significant risk factor for adenoid hypertrophy. In another study, Marek & Piotr^17^, found that allergic rhinitis can be considered a risk factor for the development of adenoid hypertrophy in children with atopic sensitization to respiratory allergens.

The frequency of adenoid hypertrophy in the mouth breathing children evaluated was 46% and tonsillar hypertrophy was 47%. Of all the patients tested, 67% had hypertrophy in at least one of the lymphoid tissues, which was considerably enlarged when compared to the normal population; but consistent with the literature when compared to the study published by Barros et al.^1^, wherein the tonsil hypertrophy frequency was 70%. Lymphoid tissue enlargement in Waldeyer's ring in children was the main cause of chronic oral breathing in the patients reported by Barros et al.^1^.

After finding a high prevalence of atopy in mouth breathers, we sought to identify a correlation between the presence and degree of tonsillar hypertrophy. No direct correlation between tonsil size and atopy was found, suggesting that other possible factors are also important in causing chronic inflammation in the non-atopic population. Carr et al.^18^ studied 117 patients with obstructive sleep apnea and adenoid hypertrophy and found no direct relationship between upper airway allergy and adenoid hypertrophy.

The high prevalence of atopy and adenotonsillar hypertrophy found in mouth breathers underscores the importance of a complete ENT and allergies assessment, because these are medical conditions in which proper treatment will bring about quality of life improvements for these patients, avoiding functional and structural changes. Multivariate analysis studies are needed to identify other risk factors directly related to adenotonsillar chronic nonallergic inflammation and its consequent hypertrophy.

## CONCLUSION

The frequency of atopy and adenotonsillar hypertrophy in the mouth breathing children evaluated in this study was higher when compared to the average found in the general population. The allergen profile in the study population was similar to those found in other Brazilian studies. There was no direct correlation between atopy and the degree of tonsils and adenoids enlargement in the mouth breathing patients evaluated.
